# Supplementary material for: Persuadable voters decided the 2022 midterm: Abortion rights and issues-based frameworks for studying election outcomes
Source: PLoS One. 2024 Jan 19;19(1):e0294047. doi: 10.1371/journal.pone.0294047 (PMC10798438; doi:10.1371/journal.pone.0294047)
Supplement: S1 Appendix — (PDF) [file pone.0294047.s001.pdf]

Supplementary Information

Persuadable voters decided the 2022 midterm: Abortion rights and issues-based frameworks for studying election outcomes

Claudia Kann, Daniel Ebanks, Jacob Morrier,
R. Michael Alvarez

A Supporting Information

A.1 Survey Question Wording

A.1.1 Vote Choice

House vote: In the November 2022 election for U.S. Congress in the district where you live, which candidate did you vote for?

- Democratic candidate
- Republican candidate
- Neither
- Not sure
- Didn't vote

A.1.2 Economics

Financial Situation: We are interested in how people are getting along financially these days. Would you say that you and your family living here are better off or worse off financially than you were a year ago?

- Better off
- The same
- Worse off

Economic Situation: Now thinking about the economy. Would you say that over the past year the nation's economy has gotten better, stayed the same, or gotten worse?

- Gotten better
- Stayed the same
- Gotten worse

A.1.3 Issues

Most Important: How important, if at all, were each of the following issues for you as you thought about whom you would vote for in the congressional election in your area in November 2022?
Rows:

- Immigration
- Abortion
- Foreign Policy
- Economic Inequality

- The COVID-19 outbreak
- Violent crime
- Health care
- The economy
- Racial and ethnic inequality
- Climate change
- Inflation
- Gun policy
- Supreme Court appointments

Columns:

- Very important
- Somewhat important
- Not too important
- Not important at all

Better Job: Which political party would do a better job with:
Rows:

- Preventing terrorism
- Mitigating climate change
- Abortion policy
- Law enforcement and criminal justice reform
- Preventing further spread of Covid-19
- Reducing the federal budget deficit
- Growing the economy
- Providing affordable healthcare
- American foreign policy
- Inflation

Columns:

- Democrat
- n
- Not sure

A.1.4 Demographics

Race: What racial or ethnic group best describes you?

- White
- Black or African American
- Hispanic or Latino
- Asian American
- American Indian/Native American
- Arab, Middle Eastern, or North African
- Native Hawaiian
- Not Hawaiian, but other Pacific Islander

Religion: What is your religious preference? Is it Protestant, Catholic, Jewish, Muslim, some other religion, or no religion?

- Protestant
- Catholic
- Jewish
- Muslim
- Some other religion
- No religion

Education: What is the highest level of education you have completed?

- No HS
- High school graduate
- Some college
- 2-year
- 4-year
- Post-grad

Age: Respondent age by category

- Under 30
- 30-44
- 54-64
- 65+

Region: Calculated from respondent's state of residence

- Northeast
- Midwest

- South
- West

Gender: Which gender identity do you most identify with?

- Woman
- Man
- Non-Binary/Fluid
- Prefer not to say

A.2 Crosstabulations

**Characteristic**	**Democrat**	**Republican**	**Neither**	**Not sure**	**Didn't vote**
Party ID					
Democrat	93% (0.01)	1.4% (0.00)	1.2% (0.00)	0.5% (0.00)	3.5% (0.01)
Republican	4.9% (0.01)	89% (0.01)	1.9% (0.01)	0.8% (0.00)	3.0% (0.01)
Independent	37% (0.02)	42% (0.02)	9.0% (0.01)	2.1% (0.01)	10% (0.01)
Other	36% (0.06)	47% (0.06)	7.1% (0.04)	3.8% (0.02)	5.9% (0.02)
Not sure	34% (0.10)	13% (0.06)	10% (0.05)	14% (0.08)	28% (0.09)
% (SE(%))					

Table A: Weighted congressional party vote choice by party ID

**Characteristic**	**Democrat**	**Republican**	**Neither**	**Not sure**	**Didn't vote**
Party ID					
Democrat	740 (93%)	14 (1.8%)	11 (1.4%)	4 (0.5%)	25 (3.1%)
Republican	24 (3.9%)	565 (91%)	10 (1.6%)	5 (0.8%)	19 (3.0%)
Independent	215 (37%)	234 (41%)	60 (10%)	11 (1.9%)	54 (9.4%)
Other	29 (36%)	38 (48%)	4 (5.0%)	3 (3.8%)	6 (7.5%)
Not sure	10 (26%)	6 (16%)	5 (13%)	4 (11%)	13 (34%)
n(unweighted) (% unweighted)					

Table B: Unweighted congressional party vote choice by party ID

**Characteristic**	**Democratic candidate**	**Republican candidate**

Gender		
Woman	54% (0.02)	46% (0.02)
Man	44% (0.02)	56% (0.02)
Non-Binary/Fluid	96% (0.04)	4.3% (0.04)
Prefer not to say	34% (0.16)	66% (0.16)
Educational Attainment		
No HS	44% (0.08)	56% (0.08)
High school graduate	44% (0.03)	56% (0.03)
Some college	50% (0.03)	50% (0.03)
2-year	51% (0.04)	49% (0.04)
4-year	50% (0.02)	50% (0.02)
Post-grad	63% (0.03)	37% (0.03)
Region		
Northeast	57% (0.03)	43% (0.03)
Midwest	46% (0.03)	54% (0.03)
South	45% (0.02)	55% (0.02)
West	57% (0.03)	43% (0.03)
Race		
White	43% (0.01)	57% (0.01)
Black or African American	84% (0.03)	16% (0.03)
Hispanic or Latino	59% (0.05)	41% (0.05)
Asian American	61% (0.09)	39% (0.09)
American Indian/Native American	37% (0.09)	63% (0.09)
Arab, Middle Eastern, or North African	77% (0.19)	23% (0.19)
Native Hawaiian	100% (0.00)	0% (0.00)
Not Hawaiian, but other Pacific Islander	65% (0.19)	35% (0.19)
Religion		
Protestant	35% (0.02)	65% (0.02)
Catholic	43% (0.03)	57% (0.03)
Jewish	67% (0.06)	33% (0.06)
Muslim	81% (0.11)	19% (0.11)
Some other religion	50% (0.03)	50% (0.03)
No religion	71% (0.02)	29% (0.02)
Age		
Under 30	63% (0.04)	37% (0.04)
30-44	54% (0.03)	46% (0.03)
45-64	43% (0.02)	57% (0.02)
65+	50% (0.02)	50% (0.02)
% (SE(%))		

Table C: Weighted demographics by party ID

Characteristic	Democratic candidate	Republican candidate
Gender		
Woman	584 (58%)	423 (42%)
Man	412 (49%)	427 (51%)
Non-Binary/Fluid	19 (95%)	1 (5.0%)
Prefer not to say	3 (33%)	6 (67%)
Educational Attainment		
No HS	23 (48%)	25 (52%)
High school graduate	191 (48%)	211 (52%)
Some college	225 (54%)	191 (46%)
2-year	118 (55%)	95 (45%)
4-year	262 (53%)	231 (47%)
Post-grad	199 (66%)	104 (34%)
Region		
Northeast	241 (61%)	153 (39%)
Midwest	208 (55%)	167 (45%)
South	311 (46%)	362 (54%)
West	258 (60%)	175 (40%)
Race		
White	696 (48%)	741 (52%)
Black or African American	185 (86%)	29 (14%)
Hispanic or Latino	91 (65%)	50 (35%)
Asian American	20 (62%)	12 (38%)
American Indian/Native American	11 (34%)	21 (66%)
Arab, Middle Eastern, or North African	6 (86%)	1 (14%)
Native Hawaiian	6 (100%)	0 (0%)
Not Hawaiian, but other Pacific Islander	3 (50%)	3 (50%)
Religion		
Protestant	237 (40%)	360 (60%)
Catholic	185 (45%)	224 (55%)
Jewish	44 (68%)	21 (32%)
Muslim	10 (77%)	3 (23%)
Some other religion	150 (55%)	122 (45%)
No religion	392 (76%)	127 (24%)
Age		
Under 30	145 (67%)	73 (33%)
30-44	195 (58%)	140 (42%)
45-64	348 (48%)	370 (52%)
65+	330 (55%)	274 (45%)

n (unweighted) (% (unweighted))

Table D: Unweighted demographics by party ID

Characteristic	Democratic candidate	Republican candidate
Immigration		
Very important	29% (0.02)	71% (0.02)
Somewhat important	65% (0.02)	35% (0.02)
Not too important	85% (0.02)	15% (0.02)
Not important at all	79% (0.05)	21% (0.05)
Abortion		
Very important	67% (0.02)	33% (0.02)
Somewhat important	51% (0.03)	49% (0.03)
Not too important	24% (0.03)	76% (0.03)
Not important at all	12% (0.02)	88% (0.02)
Foreign Policy		
Very important	42% (0.02)	58% (0.02)
Somewhat important	55% (0.02)	45% (0.02)
Not too important	62% (0.03)	38% (0.03)
Not important at all	43% (0.07)	57% (0.07)
Economic Inequality		
Very important	77% (0.02)	23% (0.02)
Somewhat important	56% (0.02)	44% (0.02)
Not too important	20% (0.03)	80% (0.03)
Not important at all	6.3% (0.01)	94% (0.01)
COVID-19		
Very important	73% (0.02)	27% (0.02)
Somewhat important	65% (0.02)	35% (0.02)
Not too important	37% (0.03)	63% (0.03)
Not important at all	11% (0.02)	89% (0.02)
Violent Crime		
Very important	36% (0.02)	64% (0.02)
Somewhat important	61% (0.02)	39% (0.02)
Not too important	81% (0.03)	19% (0.03)
Not important at all	85% (0.06)	15% (0.06)
Health Care		
Very important	68% (0.02)	32% (0.02)
Somewhat important	38% (0.02)	62% (0.02)
Not too important	19% (0.03)	81% (0.03)
Not important at all	13% (0.04)	87% (0.04)
The Economy		

Very important	38% (0.01)	62% (0.01)
Somewhat important	79% (0.02)	21% (0.02)
Not too important	90% (0.04)	10% (0.04)
Not important at all	52% (0.11)	48% (0.11)
Racial and Ethnic Inequality		
Very important	81% (0.02)	19% (0.02)
Somewhat important	59% (0.02)	41% (0.02)
Not too important	26% (0.03)	74% (0.03)
Not important at all	7.0% (0.01)	93% (0.01)
Climate Change		
Very important	84% (0.02)	16% (0.02)
Somewhat important	60% (0.03)	40% (0.03)
Not too important	23% (0.03)	77% (0.03)
Not important at all	4.7% (0.01)	95% (0.01)
Inflation		
Very important	35% (0.01)	65% (0.01)
Somewhat important	80% (0.02)	20% (0.02)
Not too important	91% (0.03)	9.2% (0.03)
Not important at all	67% (0.10)	33% (0.10)
Gun Policy		
Very important	61% (0.02)	39% (0.02)
Somewhat important	47% (0.02)	53% (0.02)
Not too important	34% (0.03)	66% (0.03)
Not important at all	18% (0.03)	82% (0.03)
Supreme Court Appointments		
Very important	60% (0.02)	40% (0.02)
Somewhat important	44% (0.02)	56% (0.02)
Not too important	34% (0.03)	66% (0.03)
Not important at all	20% (0.04)	80% (0.04)

% (SE(%))

Table E: Weighted most important issue by party ID

**Characteristic**	**Democratic candidate**	**Republican candidate**
Immigration		
Very important	286 (31%)	635 (69%)
Somewhat important	402 (70%)	174 (30%)
Not too important	259 (88%)	35 (12%)
Not important at all	70 (84%)	13 (16%)
Abortion		
Very important	737 (72%)	289 (28%)

Somewhat important	184 (53%)	160 (47%)
Not too important	62 (25%)	182 (75%)
Not important at all	35 (13%)	226 (87%)
Foreign Policy		
Very important	336 (46%)	398 (54%)
Somewhat important	481 (59%)	339 (41%)
Not too important	172 (66%)	89 (34%)
Not important at all	29 (48%)	31 (52%)
Economic Inequality		
Very important	603 (81%)	140 (19%)
Somewhat important	328 (61%)	214 (39%)
Not too important	64 (22%)	230 (78%)
Not important at all	23 (7.8%)	273 (92%)
COVID-19		
Very important	438 (78%)	124 (22%)
Somewhat important	378 (68%)	175 (32%)
Not too important	146 (40%)	220 (60%)
Not important at all	54 (14%)	338 (86%)
Violent Crime		
Very important	407 (39%)	640 (61%)
Somewhat important	346 (67%)	174 (33%)
Not too important	203 (85%)	36 (15%)
Not important at all	62 (90%)	7 (10%)
Health Care		
Very important	722 (72%)	275 (28%)
Somewhat important	246 (40%)	362 (60%)
Not too important	38 (19%)	157 (81%)
Not important at all	12 (16%)	63 (84%)
The Economy		
Very important	531 (41%)	757 (59%)
Somewhat important	389 (82%)	83 (18%)
Not too important	83 (91%)	8 (8.8%)
Not important at all	15 (62%)	9 (38%)
Racial and Ethnic Inequality		
Very important	588 (85%)	106 (15%)
Somewhat important	306 (62%)	188 (38%)
Not too important	93 (28%)	245 (72%)
Not important at all	31 (8.9%)	318 (91%)
Climate Change		
Very important	665 (88%)	95 (12%)
Somewhat important	267 (65%)	144 (35%)
Not too important	67 (25%)	206 (75%)

Not important at all	19 (4.4%)	412 (96%)
Inflation		
Very important	466 (38%)	767 (62%)
Somewhat important	355 (84%)	69 (16%)
Not too important	169 (93%)	13 (7.1%)
Not important at all	27 (77%)	8 (23%)
Gun Policy		
Very important	657 (66%)	339 (34%)
Somewhat important	243 (51%)	237 (49%)
Not too important	84 (35%)	154 (65%)
Not important at all	34 (21%)	126 (79%)
Supreme Court Appointments		
Very important	666 (65%)	365 (35%)
Somewhat important	250 (47%)	282 (53%)
Not too important	80 (37%)	136 (63%)
Not important at all	22 (23%)	74 (77%)

n (unweighted) (% (unweighted))

Table F: Unweighted most important issue by party ID

**Characteristic**	**Democratic candidate**	**Republican candidate**
Preventing Terrorism		
Democrat	91% (0.01)	8.5% (0.01)
Republican	13% (0.01)	87% (0.01)
Not sure	75% (0.03)	25% (0.03)
Mitigating Climate Change		
Democrat	84% (0.01)	16% (0.01)
Republican	14% (0.02)	86% (0.02)
Not sure	22% (0.02)	78% (0.02)
Abortion Policy		
Democrat	87% (0.01)	13% (0.01)
Republican	10.0% (0.01)	90% (0.01)
Not sure	29% (0.03)	71% (0.03)
Criminal justice reform		
Democrat	93% (0.01)	7.3% (0.01)
Republican	12% (0.01)	88% (0.01)
Not sure	65% (0.03)	35% (0.03)
COVID-19		
Democrat	90% (0.01)	9.6% (0.01)
Republican	11% (0.02)	89% (0.02)
Not sure	28% (0.02)	72% (0.02)

Deficit		
Democrat	93% (0.01)	6.5% (0.01)
Republican	14% (0.01)	86% (0.01)
Not sure	63% (0.03)	37% (0.03)
The Economy		
Democrat	92% (0.01)	7.7% (0.01)
Republican	11% (0.01)	89% (0.01)
Not sure	75% (0.03)	25% (0.03)
Health Care		
Democrat	87% (0.01)	13% (0.01)
Republican	11% (0.02)	89% (0.02)
Not sure	22% (0.02)	78% (0.02)
Foreign Policy		
Democrat	94% (0.01)	6.2% (0.01)
Republican	11% (0.01)	89% (0.01)
Not sure	63% (0.03)	37% (0.03)
Inflation		
Democrat	91% (0.01)	9.0% (0.01)
Republican	12% (0.01)	88% (0.01)
Not sure	82% (0.02)	18% (0.02)
% (SE(%))		

Table G: Weighted ability by party ID

**Characteristic**	**Democratic candidate**	**Republican candidate**
Preventing Terrorism		
Democrat	632 (94%)	43 (6.4%)
Republican	115 (13%)	737 (87%)
Not sure	271 (78%)	77 (22%)
Mitigating Climate Change		
Democrat	851 (86%)	135 (14%)
Republican	66 (14%)	402 (86%)
Not sure	101 (24%)	320 (76%)
Abortion Policy		
Democrat	872 (89%)	112 (11%)
Republican	73 (11%)	578 (89%)
Not sure	73 (30%)	167 (70%)
Criminal justice reform		
Democrat	734 (94%)	47 (6.0%)
Republican	113 (13%)	734 (87%)
Not sure	171 (69%)	76 (31%)

COVID-19		
Democrat	823 (92%)	69 (7.7%)
Republican	57 (11%)	483 (89%)
Not sure	138 (31%)	305 (69%)
Deficit		
Democrat	652 (95%)	32 (4.7%)
Republican	129 (16%)	697 (84%)
Not sure	237 (65%)	128 (35%)
The Economy		
Democrat	749 (94%)	46 (5.8%)
Republican	108 (12%)	761 (88%)
Not sure	161 (76%)	50 (24%)
Health Care		
Democrat	885 (89%)	108 (11%)
Republican	58 (10%)	510 (90%)
Not sure	75 (24%)	239 (76%)
Foreign Policy		
Democrat	757 (95%)	41 (5.1%)
Republican	100 (12%)	734 (88%)
Not sure	161 (66%)	82 (34%)
Inflation		
Democrat	667 (93%)	50 (7.0%)
Republican	120 (14%)	759 (86%)
Not sure	231 (83%)	48 (17%)

n (unweighted) (% (unweighted))

Table H: Unweighted ability by party ID

**Characteristic**	**Democratic candidate**	**Republican candidate**
Economic Situation		
Gotten better	82% (0.03)	18% (0.03)
Stayed the same	80% (0.02)	20% (0.02)
Gotten worse	34% (0.01)	66% (0.01)
Financial Situation		
Better off	70% (0.03)	30% (0.03)
The same	65% (0.02)	35% (0.02)
Worse off	30% (0.02)	70% (0.02)

% (SE(%))

Table I: Weighted views on financial situation by party ID

Characteristic	Democratic candidate	Republican candidate
Economic Situation		
Gotten better	212 (89%)	27 (11%)
Stayed the same	336 (84%)	65 (16%)
Gotten worse	470 (38%)	765 (62%)
Financial Situation		
Better off	193 (76%)	62 (24%)
The same	545 (69%)	240 (31%)
Worse off	280 (34%)	555 (66%)
n (unweighted) (% (unweighted))		

Table J: Unweighted views on financial situation by party

A.3 Regression Result Figures

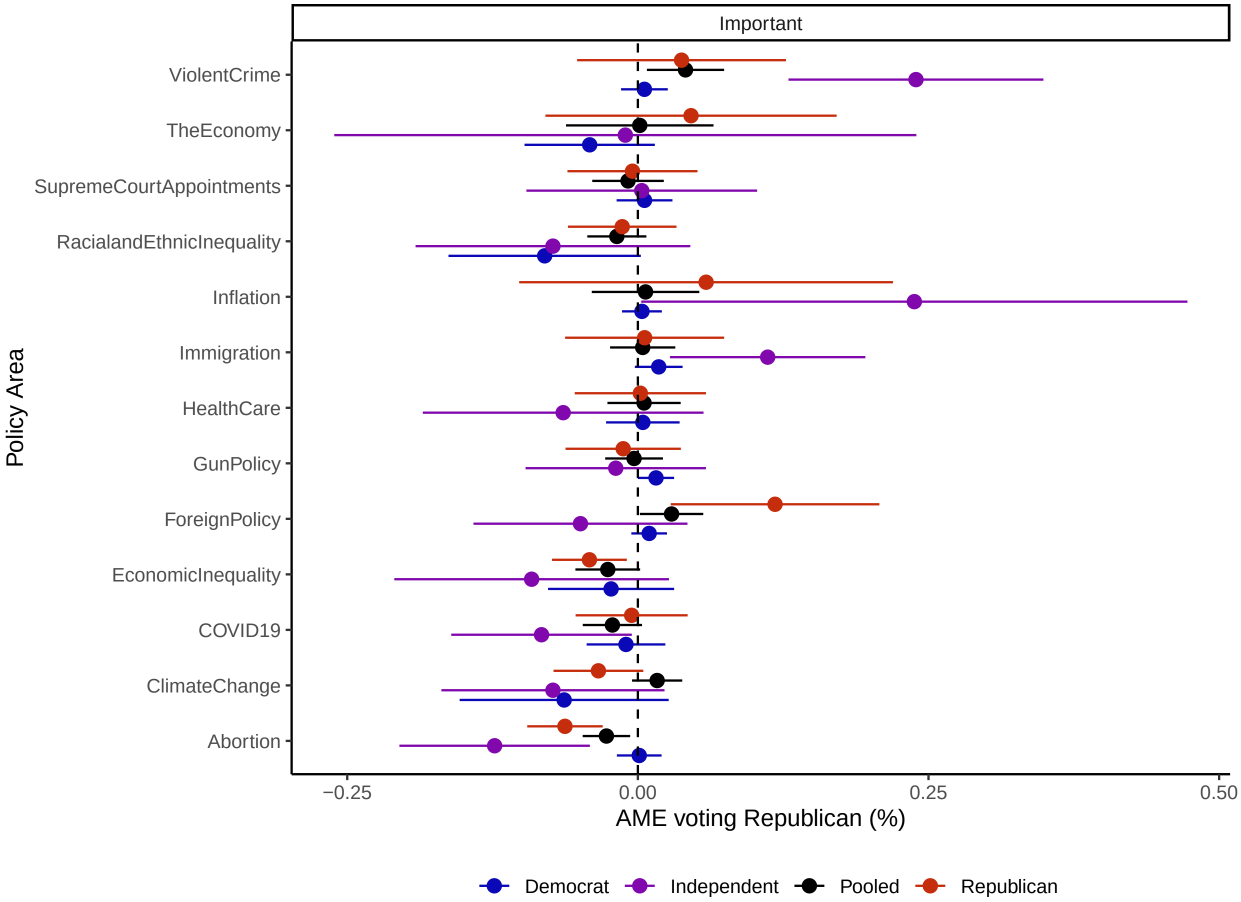

Fig A: Average marginal effect (with 95% confidence intervals) of viewing policy areas as important on the probability of voting for the Republican congressional candidate. The plots show the results for the party-based model for each party as well as the pooled model results.

A.4 Regression Result Tables

	Average Marginal Effects:			
	Probability Voting for Republican for Congress			
	Full	Model 1	Model 2	Model 3
**Economics**				
Follow Goverment				

Don't know	0.106**	0.124***	0.118**	
	(0.041)	(0.048)	(0.053)	
Hardly at all	0.025*	0.027	0.027*	
	(0.014)	(0.017)	(0.016)	
Most of the time	−0.012	−0.015	−0.015	
	(0.012)	(0.011)	(0.011)	
Only now and then	0.001	0.002	0.004	
	(0.013)	(0.014)	(0.014)	
EconomicSituation				
Gotten better	0.027	0.025	0.023	
	(0.026)	(0.019)	(0.021)	
Gotten worse	0.031**	0.033**	0.032**	
	(0.015)	(0.014)	(0.014)	
Financial Situation				
Better off	0.002			
	(0.019)			
Worse off	0.002			
	(0.011)			
**Party Abilities**				
Health Care				
Democrat	−0.058***	−0.064***	−0.059***	−0.146***
	(0.016)	(0.019)	(0.018)	(0.025)
Republican	−0.053***	−0.057***	−0.052***	−0.034
	(0.014)	(0.017)	(0.016)	(0.022)
Inflation				
Democrat	0.105***	0.076**	0.075**	0.049
	(0.039)	(0.031)	(0.029)	(0.034)
Republican	0.094**	0.097***	0.098***	0.180***
	(0.044)	(0.031)	(0.029)	(0.033)
Abortion				
Democrat	−0.044**	−0.066***	−0.062***	
	(0.018)	(0.019)	(0.019)	
Republican	0.029*	0.035	0.038	
	(0.016)	(0.022)	(0.024)	
Covid				
Democrat	−0.047**	−0.063**	−0.064***	
	(0.024)	(0.026)	(0.023)	
Republican	−0.004	−0.006	−0.012	
	(0.014)	(0.016)	(0.015)	
Crime				
Democrat	0.035**	0.027	0.023	

	(0.016)	(0.019)	(0.020)	
Republican	0.018	0.027*	0.026*	
	(0.016)	(0.014)	(0.014)	
Budget				
Democrat	−0.025			
	(0.022)			
Republican	−0.001			
	(0.015)			
Foreign Policy				
Democrat	−0.001			
	(0.020)			
Republican	0.004			
	(0.017)			
Climate				
Democrat	−0.015			
	(0.014)			
Republican	−0.017			
	(0.012)			
Grow Economy				
Democrat	−0.028			
	(0.025)			
Republican	−0.00004			
	(0.017)			
Law Enforcement				
Democrat	−0.021			
	(0.023)			
Republican	0.018			
	(0.017)			
**Important Issues**				
Abortion	−0.032***	−0.036***	−0.038***	−0.068***
	(0.011)	(0.012)	(0.012)	(0.014)
Foreign Policy	0.035***	0.032***	0.025**	0.022
	(0.013)	(0.011)	(0.012)	(0.017)
Economic Inequality	−0.026**	−0.046***	−0.046***	
	(0.013)	(0.013)	(0.014)	
Violent Crime	0.041**	0.041***	0.039**	
	(0.016)	(0.015)	(0.016)	
Climate Change	0.018*	0.015		
	(0.011)	(0.012)		
COVID19	−0.023*	−0.025*		
	(0.014)	(0.014)		

Gun Policy	−0.005			
	(0.013)			
Health Care	0.006			
	(0.015)			
Immigration	0.005			
	(0.013)			
Inflation	0.003			
	(0.022)			
Racial and Ethnic Inequality	−0.016			
	(0.012)			
Supreme Court Appointments	−0.003			
	(0.015)			
The Economy	0.004			
	(0.029)			
**Demographics**				
PartyID				
Democrat	-0.239^{***}	-0.258^{***}	-0.261^{***}	-0.320^{***}
	(0.041)	(0.040)	(0.039)	(0.039)
Not sure	−0.080	-0.120^{**}	-0.122^{**}	-0.117^{**}
	(0.063)	(0.061)	(0.060)	(0.059)
Other	−0.014	−0.013	−0.006	−0.011
	(0.029)	(0.030)	(0.030)	(0.033)
Republican	0.158^{***}	0.174^{***}	0.171^{***}	0.246^{***}
	(0.032)	(0.032)	(0.033)	(0.036)
Race				
American Indian/Native American	-0.053^{*}	-0.050^{*}	-0.046^{*}	−0.056
	(0.030)	(0.027)	(0.028)	(0.034)
Arab, Middle Eastern, or North African	-0.103^{*}	-0.103^{**}	-0.119^{**}	-0.107^{**}
	(0.058)	(0.051)	(0.056)	(0.051)
Asian American	0.046	0.044^{*}	0.040	0.022
	(0.029)	(0.027)	(0.026)	(0.024)
Black or African American	-0.044^{**}	-0.053^{***}	-0.056^{***}	-0.053^{**}
	(0.020)	(0.019)	(0.020)	(0.026)
Hispanic or Latino	−0.001	−0.001	−0.005	0.007
	(0.014)	(0.016)	(0.016)	(0.021)
Native Hawaiian	-0.501^{***}	-0.503^{***}	-0.505^{***}	-0.505^{***}
	(0.006)	(0.006)	(0.006)	(0.006)

Not Hawaiian, but other Pacific Islander	0.056	0.058	0.055	−0.047
	(0.104)	(0.091)	(0.084)	(0.113)
Region				
Midwest	0.0003	0.0004	−0.001	0.003
	(0.016)	(0.016)	(0.015)	(0.016)
South	0.013	0.013	0.015	0.025
	(0.013)	(0.013)	(0.013)	(0.015)
West	0.002	−0.001	0.001	0.008
	(0.014)	(0.013)	(0.013)	(0.017)
Religion				
Catholic	0.003	0.002	0.003	−0.005
	(0.011)	(0.013)	(0.013)	(0.017)
Jewish	−0.053*	−0.048*	−0.053*	−0.069**
	(0.031)	(0.026)	(0.029)	(0.035)
Muslim	0.055*	0.059**	0.058**	0.017
	(0.028)	(0.028)	(0.028)	(0.036)
No religion	−0.013	−0.015	−0.015	−0.040***
	(0.012)	(0.012)	(0.012)	(0.015)
Some other religion	−0.009	−0.005	−0.004	−0.005
	(0.016)	(0.017)	(0.017)	(0.019)
Age				
30-44	0.018	0.017	0.018	0.007
	(0.012)	(0.011)	(0.011)	(0.014)
45-64	0.009	0.007	0.009	0.018
	(0.012)	(0.011)	(0.012)	(0.012)
Under 30	−0.023	−0.031*	−0.030*	−0.024
	(0.017)	(0.017)	(0.018)	(0.022)
Education				
College Graduate	−0.011	−0.013	−0.011	−0.020*
	(0.010)	(0.010)	(0.010)	(0.010)
Post Grad	−0.020	−0.027	−0.025	−0.038*
	(0.020)	(0.020)	(0.021)	(0.021)
Gender				
Non-Binary/Fluid	−0.022	−0.038	−0.050	−0.029
	(0.071)	(0.072)	(0.082)	(0.063)
Prefer not to say	0.058**	0.059**	0.056**	0.095*
	(0.024)	(0.025)	(0.025)	(0.049)
Woman	−0.006	−0.006	−0.006	−0.010
	(0.009)	(0.009)	(0.009)	(0.010)
Observations	1,871	1,871	1,871	1,871

Note:	*p<0.1; **p<0.05; ***p<0.01

Table K: Comparison of pooled models

	Dependent variable:			
	Congressional Vote			
	Pooled	Republican	Democrat	Independent
**Economics**				
EconomicSituation				
Gotten better	0.023	0.019	-0.009^{***}	-0.502^{***}
	(0.021)	(0.021)	(0.0001)	(0.025)
Gotten worse	0.032^{**}	0.019	0.031^{***}	0.050^{*}
	(0.014)	(0.019)	(0.001)	(0.029)
FollowGoverment				
Don't know	0.118^{**}	0.031^{***}	0.102^{***}	-0.516^{***}
	(0.053)	(0.008)	(0.003)	(0.018)
Hardly at all	0.027^{*}	0.016	0.003^{***}	0.120^{**}
	(0.016)	(0.012)	(0.001)	(0.048)
Most of the time	-0.015	-0.026	-0.027^{***}	0.008
	(0.011)	(0.017)	(0.001)	(0.022)
Only now and then	0.004	-0.013	-0.035^{***}	0.059
	(0.014)	(0.017)	(0.001)	(0.051)
**Party Abilities**				
Abortion				
Democrat	-0.062^{***}	-0.064	-0.065^{***}	-0.049
	(0.019)	(0.048)	(0.002)	(0.047)
Republican	0.038	0.017	0.013^{***}	0.131^{***}
	(0.024)	(0.030)	(0.002)	(0.049)
Covid				
Democrat	-0.064^{***}	-0.062^{***}	-0.066^{***}	-0.146^{***}
	(0.023)	(0.023)	(0.002)	(0.050)
Republican	-0.012	-0.003	-0.080^{***}	-0.075^{*}
	(0.015)	(0.015)	(0.002)	(0.043)
Crime				
Democrat	0.023	0.034	-0.000	0.118^{***}
	(0.020)	(0.049)	(0.000)	(0.037)
Republican	0.026^{*}	0.036	-0.022^{***}	0.066^{**}
	(0.014)	(0.048)	(0.00000)	(0.032)
HealthCare				
Democrat	-0.059^{***}	-0.046^{*}	0.0005^{**}	-0.064

	(0.018)	(0.023)	(0.0002)	(0.047)
Republican	−0.052***	−0.077***	0.176***	−0.012
	(0.016)	(0.018)	(0.004)	(0.032)
Inflation				
Democrat	0.075**	0.189	0.012***	−0.002
	(0.029)	(0.183)	(0.003)	(0.048)
Republican	0.098***	0.210	0.023***	0.135***
	(0.029)	(0.184)	(0.001)	(0.046)
**Important Issues**				
Abortion	−0.038***	−0.020	−0.00004	−0.110***
	(0.012)	(0.015)	(0.0001)	(0.038)
EconomicInequality	−0.046***	−0.041***	−0.083***	−0.062*
	(0.014)	(0.012)	(0.002)	(0.033)
ForeignPolicy	0.025**	0.053*	0.017***	−0.003
	(0.012)	(0.031)	(0.00000)	(0.027)
ViolentCrime	0.039**	0.041	−0.014**	0.115**
	(0.016)	(0.032)	(0.006)	(0.048)
**Demographics**				
PartyID				
Democrat	−0.261***			
	(0.039)			
Not sure	−0.122**			
	(0.060)			
Other	−0.006			
	(0.030)			
Republican	0.171***			
	(0.033)			
Age				
30-44	0.018	0.015	0.009***	0.053*
	(0.011)	(0.023)	(0.003)	(0.029)
45-64	0.009	−0.009	−0.011***	0.039
	(0.012)	(0.023)	(0.002)	(0.029)
Under 30	−0.030*	−0.046	−0.021***	−0.005
	(0.018)	(0.034)	(0.001)	(0.038)
Education				
College Graduate	−0.011	−0.018	0.032***	−0.001
	(0.010)	(0.021)	(0.001)	(0.023)
Post Grad	−0.025	−0.053	0.051***	−0.031
	(0.021)	(0.033)	(0.002)	(0.030)
Gender				
Non-Binary/Fluid	−0.050		0.161***	−0.040
	(0.082)		(0.006)	(0.070)

Prefer not to say	0.056**		0.277***	0.086
	(0.025)		(0.008)	(0.059)
Woman	−0.006	0.008	0.023***	−0.059**
	(0.009)	(0.014)	(0.001)	(0.024)
Race				
American Indian/Native American	−0.046*	−0.042	−0.028***	−0.104*
	(0.028)	(0.030)	(0.001)	(0.062)
Arab, Middle Eastern, or North African	−0.119**	0.037***	−0.001	−0.543***
	(0.056)	(0.005)	(0.001)	(0.014)
Asian American	0.040	0.037***	0.041***	0.178***
	(0.026)	(0.005)	(0.007)	(0.054)
Black or African American	−0.056***	−0.031	−0.019***	−0.105*
	(0.020)	(0.028)	(0.002)	(0.057)
Hispanic or Latino	−0.005	−0.027	−0.023***	0.017
	(0.016)	(0.032)	(0.001)	(0.028)
Native Hawaiian	−0.505***		−0.010***	−0.536***
	(0.006)		(0.004)	(0.014)
Not Hawaiian, but other Pacific Islander	0.055	−0.084	0.714***	−0.097
	(0.084)	(0.053)	(0.007)	(0.070)
Region				
Midwest	−0.001	−0.003	−0.014***	0.021
	(0.015)	(0.028)	(0.002)	(0.038)
South	0.015	0.012	−0.026***	0.052
	(0.013)	(0.021)	(0.002)	(0.037)
West	0.001	−0.009	0.035***	0.025
	(0.013)	(0.023)	(0.001)	(0.039)
Religion				
Catholic	0.003	−0.010	−0.002**	0.082**
	(0.013)	(0.014)	(0.001)	(0.041)
Jewish	−0.053*	−0.056	−0.005***	−0.086
	(0.029)	(0.062)	(0.002)	(0.061)
Muslim	0.058**	0.013	0.089***	0.402***
	(0.028)	(0.020)	(0.005)	(0.022)
No religion	−0.015	−0.006	−0.018***	0.006
	(0.012)	(0.022)	(0.001)	(0.026)
Some other religion	−0.004	−0.002	0.030***	0.002
	(0.017)	(0.021)	(0.005)	(0.039)
Observations	1,871	588	751	449

Note:	*p$<$0.1; **p$<$0.05; ***p$<$0.01

Table L: Final regression results

A.5 Pooling Tests

Variable	Wald Statistic
Abortion	118.85
COVID19	1701.37
ClimateChange	1349.41
Economic Situation: Gotten better	3139.38
Economic Situation: Gotten worse	2934.48
EconomicInequality	788.16
Financial Situation: Better off	1232.64
Financial Situation: Worse off	1686.93
ForeignPolicy	2773.38
GunPolicy	897.08
HealthCare	1989.29
Immigration	1963.12
Inflation	4000.10
RacialandEthnicInequality	1460.28
SupremeCourtAppointments	1140.09
TheEconomy	835.61
ViolentCrime	908.58

Table M: We report Wald statistics testing whether party-level interactions are jointly 0 in a pooled model. Large test statistics suggest we can reject the null that coefficients desegregated in the pooled are jointly 0. We instead report the Average Marginal Effects from logit specifications dis-aggregated by party ID

A.6 Re-coded Party ID: Robustness

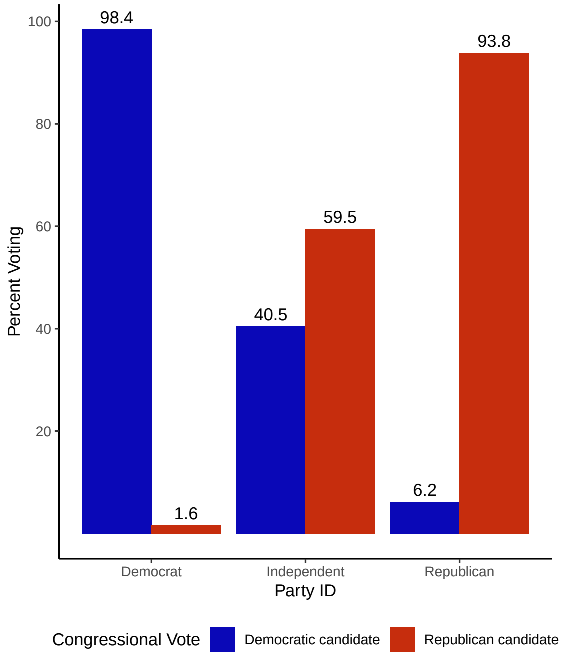

Fig B: This figure shows the weighted percentage of members of each party who voted for congressional candidates of each party. From this figure it is clear that party cohesion was strong — most people voted with their party. This supports the idea that most of the interesting variation will come from those who identify as Independents. Independents as a group make up about half as many individuals as Democrats or Republicans, indicating that they could be the swing vote in the election. Democrats were significantly more loyal to their party than Republicans.

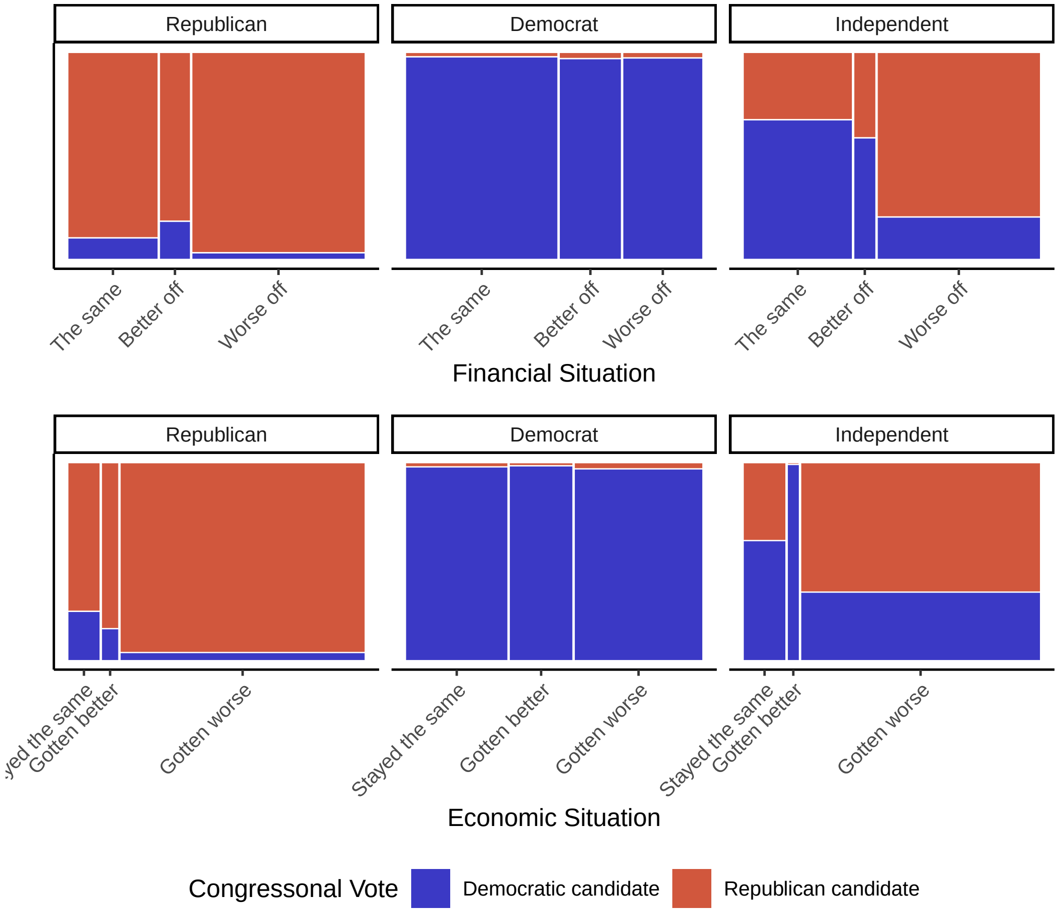

Fig C: Vote choice by recoded party identification where independents exclude leaners and by views of the national economy and personal finances as compared to the previous year. The width of the bars represent what proportion of individuals (weighted) fell into each grouping. This helps to see how the answers are related to vote choice but also how party identification relates to the response.

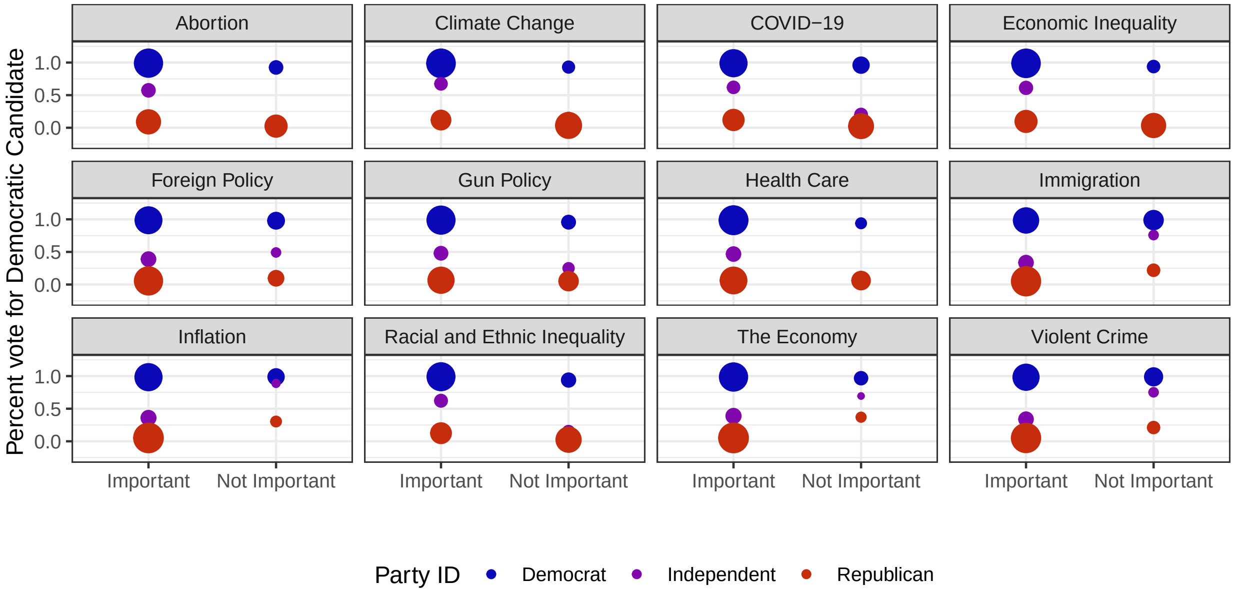

Fig D: For issues, how different party identifiers under recoded labels where Independents exclude leaners voted based on whether they thought it was important or not. The color represents partisan identification while the size is the weighted number of individuals who fit the category. It is clear that most partisans stuck to their party, regardless of their views on issues. Independents were swayed by the issues they viewed as important.

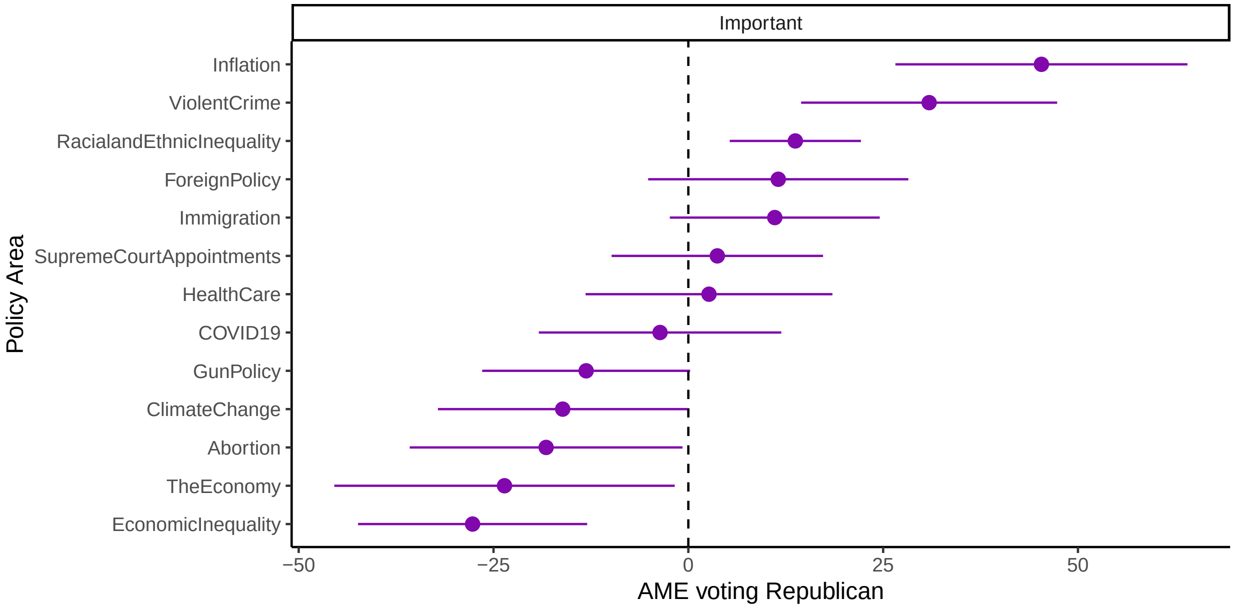

Fig E: Average marginal effect (with 95% confidence intervals) of viewing policy areas as important on the probability of voting for the Republican congressional candidate. The plots show the results for the party-based model for Independents excluding leaners.

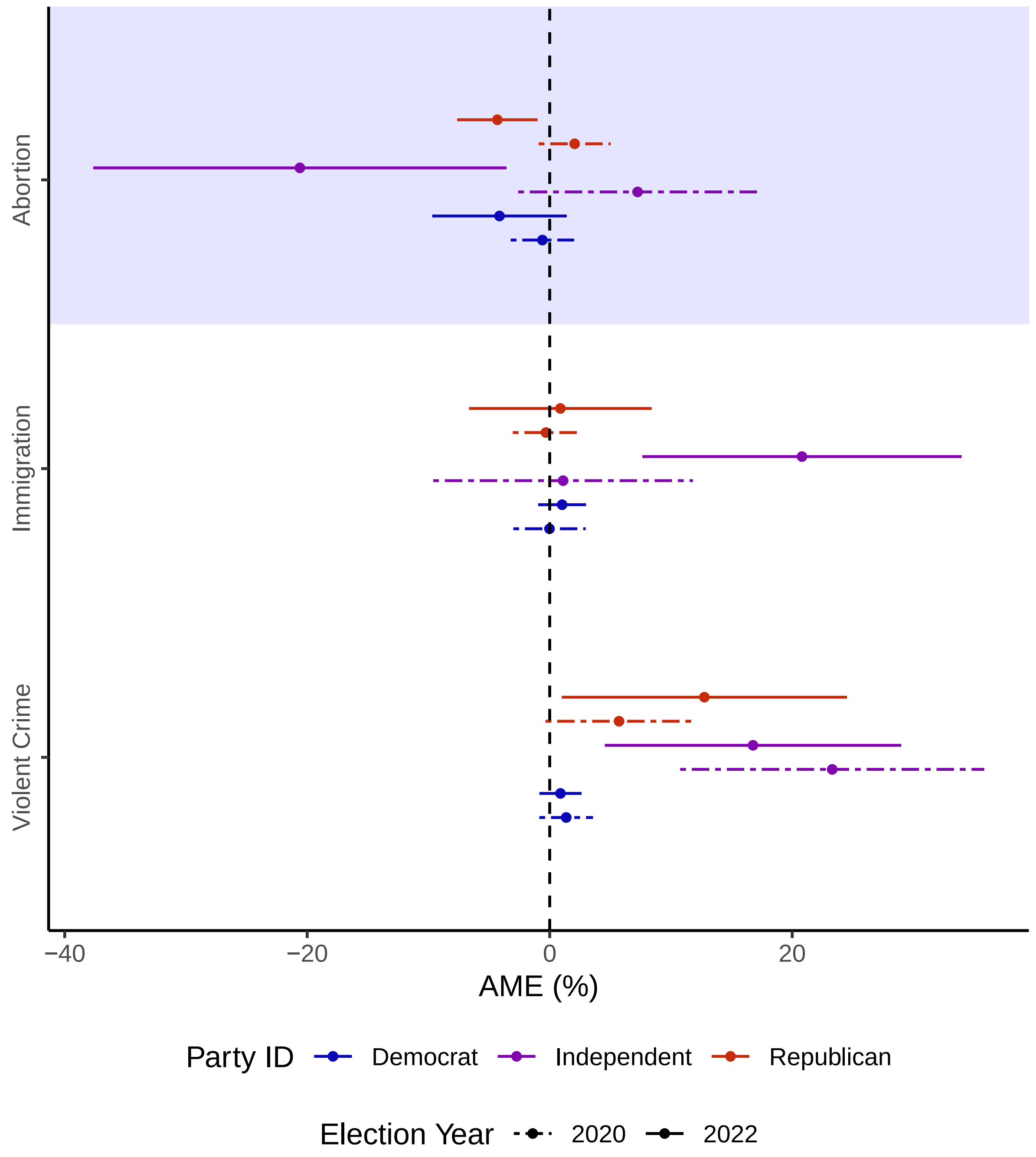

Fig F: Average marginal effect from thinking abortion, immigration, and violent crime were important on the probability of voting Republican for the 2020 and 2022 surveys. There is a clear break in the relationship between importance attributed to abortion and voting decisions for Independents (excluding leaners) and Republicans. There is no statistically significant break for any partisans for immigration or violent crime.
